# Positive allosteric modulation of AMPA receptors via PF4778574 leads to reduced demyelination and clinical disability in experimental models of multiple sclerosis

**DOI:** 10.3389/fimmu.2025.1532877

**Published:** 2025-03-05

**Authors:** Mustafa Sindi, Michael Dietrich, Diana Klees, Joel Gruchot, Christina Hecker, John Silbereis, Andrea Issberner, Hans-Peter Hartung, Tobias Ruck, Holger Stark, Thomas Kurz, Patrick Küry, Sven G. Meuth, Philipp Albrecht

**Affiliations:** ^1^ Medical Faculty and University Hospital Düsseldorf, Department of Neurology, Heinrich Heine University, Düsseldorf, Germany; ^2^ Department of Medical Research, Multiple Sclerosis Unit, Biogen, Cambridge, MA, United States; ^3^ Brain and Mind Center, University of Sydney, Sydney, NSW, Australia; ^4^ Department of Neurology, Palacky University Olomouc, Olomouc, Czechia; ^5^ Faculty of Mathematics and Natural Sciences, Institute of Pharmaceutical and Medicinal Chemistry, Heinrich Heine University, Düsseldorf, Germany; ^6^ Department of Neurology, Maria Hilf Clinics, Mönchengladbach, Germany

**Keywords:** AMPA-PAM, AMPA, EAE, excitotoxicity, neuroprotection, multiple sclerosis, optical coherence tomography

## Abstract

**Introduction:**

Multiple Sclerosis (MS), a debilitating central nervous system (CNS) disorder, is characterized by inflammation, demyelination, and neuronal degeneration. Despite advancements in immunomodulatory treatments, neuroprotective or restorative strategies remain inadequate. Our research is focusing on the potential of the positive allosteric modulator of AMPA receptors (AMPA-PAM), PF4778574, in addressing MS symptoms.

**Methods:**

We utilized the MOG35-55 induced experimental autoimmune encephalomyelitis (EAE) model in C57BL6J mice to examine PF4778574’s therapeutic and prophylactic efficacy. Our comprehensive approach included clinical scoring, optical coherence tomography (OCT), optomotor response (OMR) and histological assessments. Additionally, we explored the effects of PF4778574 in comparison and in combination with the immunomodulatory agent fingolimod, and investigated the impact on Cuprizone induced toxic demyelination.

**Results:**

Prophylactic administration of PF4778574 showed notable improvement in clinical EAE indices and reduction in neuronal loss. While it did not diminish microglial activity, it reduced demyelinated areas in optic nerves and in the corpus callosum. Both PF4778574 and fingolimod significantly enhanced clinical EAE scores and decreased demyelination. However, their combination did not yield additional benefits. In the cuprizone model, PF4778574 increased oligodendrocyte precursor and mature myelin-forming cells, suggesting a pro-remyelinating effect.

**Discussion:**

PF4778574 demonstrates promise in mitigating EAE effects, especially in terms of clinical disability and demyelination. These results suggest AMPA-PAMs as potential targets of interest for MS treatment beyond immunomodulatory approaches.

## 1 Introduction

Multiple Sclerosis (MS) is an autoimmune disorder of the central nervous system marked by inflammation and demyelination, leading to the dysfunction of oligodendrocytes, subsequent harm to axons, and neuronal degeneration in the central nervous system (1). The chronic neurodegeneration is the main reason for the sustained clinical impairment in patients with MS and is not addressable to traditional immunosuppressants and immunomodulatory treatments. Hallmarks of MS include pathological changes marked by lymphocytes and macrophages penetrating the CNS tissue, leading to the accumulation of adhesion molecules and the promotion of immune cell engagement through pro-inflammatory cytokines. These series of events results in glial damage and neuronal injury. Although the precise underlying process is yet to be completely understood, recent studies have suggested that enhancing axonal and synaptic function in MS can reduce symptoms and potentially even have protective capacities (2, 3).

AMPA-type ionotropic glutamate receptor positive allosteric modulators (AMPA-PAMs) are considered as synaptic enhancers and have been recognized for their potential in the therapeutic management of cognitive and mood disorders (4–6). These agents modulate the function of AMPA receptors, which are central to fast synaptic transmission in the nervous system. By enhancing the activity of these receptors, AMPA-PAMs facilitate various cognitive processes and could have considerable therapeutic implications (7). AMPA-PAMs bind to the AMPA receptor (AMPAR) complex’s allosteric sites, boosting the receptor. However, unlike AMPAR agonists, PAMs only enhance signals when glutamate is present, but do not directly trigger the receptor. They can also selectively target specific AMPAR subgroups (8, 9). AMPA-PAMs are divided into two types; low-impact PAMs, which minimally affect the field excitatory postsynaptic potential (fEPSP), and high-impact PAMs, which noticeably alter the fEPSP. Only high-impact PAMs, such as the compound PF4778574 tested in this study bind to the cyclothiazide site and trigger BDNF expression. Low-impact PAMs boost synaptic currents by reducing AMPAR deactivation, while high-impact PAMs enhance and extend synaptic currents by decreasing both deactivation and desensitization (10).

MS, particularly in its chronic phases, can be considered a neurodegenerative disease due to the irreversible loss of neurons and associated cognitive decline (11). At the cellular level, glutamate-induced excitotoxicity is known to contribute significantly to the neuronal damage observed in MS (12). AMPA receptors, being the primary mediators of fast excitatory synaptic transmission, are thought to play a substantial role in this process. Over-activation of AMPA receptors leads to an excessive influx of calcium ions, triggering a cascade of harmful intracellular processes that culminate in neuronal death. AMPA-PAMs, however, modulate the receptor’s function to enhance synaptic transmission without causing receptor over-activation and the ensuing excitotoxicity (6). This modulation, in essence, could aid in maintaining neuronal communication while preventing harmful consequences to the neurons. In addition, beyond their role in neurotransmission, AMPA receptors have also been implicated in inflammatory processes. Studies suggest that AMPA receptor antagonists can reduce the release of pro-inflammatory cytokines (13–15), implying that modulating AMPA receptor function might have potential anti-inflammatory effects. While the therapeutic effects of AMPA-PAMs have neither been evaluated in MS nor in experimental autoimmune encephalomyelitis (EAE), these mechanistic insights provide a rationale for further investigation. Moreover, the possibility of modulating neuroinflammation and excitotoxicity, two central processes in MS, suggests that AMPA-PAMs could also influence disease progression.

In this study, we investigated whether AMPA-PAM can help protect against neurodegeneration and demyelination—two major pathologies of MS alongside neuroinflammation. We employed the MOG35–55-induced EAE model to assess both prophylactic and therapeutic administration of AMPA-PAM alone or in combination with fingolimod, a well-established immunomodulatory drug. By integrating clinical scoring, optical coherence tomography (OCT), optomotor response (OMR), and comprehensive histological analyses, we aimed to determine if AMPA-PAM could preserve neuronal structures and myelin integrity in the setting of autoimmune inflammation. We further extended our approach to the cuprizone model to explore whether AMPA-PAM might promote remyelination under toxic demyelinating conditions. We hypothesized that AMPA-PAM would provide neuroprotection and could potentially synergize with classical immunomodulatory strategies. Although existing MS therapies effectively target inflammation, they often fail to prevent ongoing neurodegeneration. By focusing on the positive modulation of AMPA receptors, our study addresses a critical gap in current MS research investigating whether synaptic enhancement can confer neuroprotective and pro-remyelinating benefits. Ultimately, our findings may offer novel insights that help expand future therapeutic approaches beyond immunosuppression, with the aim of preserving and restoring neural function in MS.

Given these considerations, the role of AMPA-PAMs in MS warrants further investigation. This could potentially lead to therapeutic strategies not only for MS but also for a broader range of inflammatory and neurodegenerative diseases where excitotoxicity and axonal loss are involved. As such, this line of research holds promise in contributing to our understanding and treatment of neuroinflammatory and neurodegenerative diseases.

## 2 Materials and methods

### 2.1 Subject animals, models and treatment protocols

EAE induction: Six-week-old female C57BL/6J mice, sourced from Janvier Labs (Le Genest-Saint-Isle, France), were utilized for the experiment. The EAE mouse model involved immunization of mice with 200 μg of myelin oligodendrocyte glycoprotein fragment 35–55 (MOG35–55), which was acquired from BIOTREND. The immunization was emulsified in 200 μl of complete Freund’s adjuvant (CFA) and augmented with 800 μg of thermally inactivated Mt., H37Ra. Both of these components were procured from BD Difco. The immunization process included subcutaneous injection distributed over four points on the front and hind flank. Additionally, intraperitoneal injections of 200 ng of pertussis toxin (PTX), obtained from Sigma-Aldrich, were administered on days 0 and 2 post-immunization. The sham control group was treated with PTX and CFA, excluding the MOG35–55 peptide.

**Figure 1:**
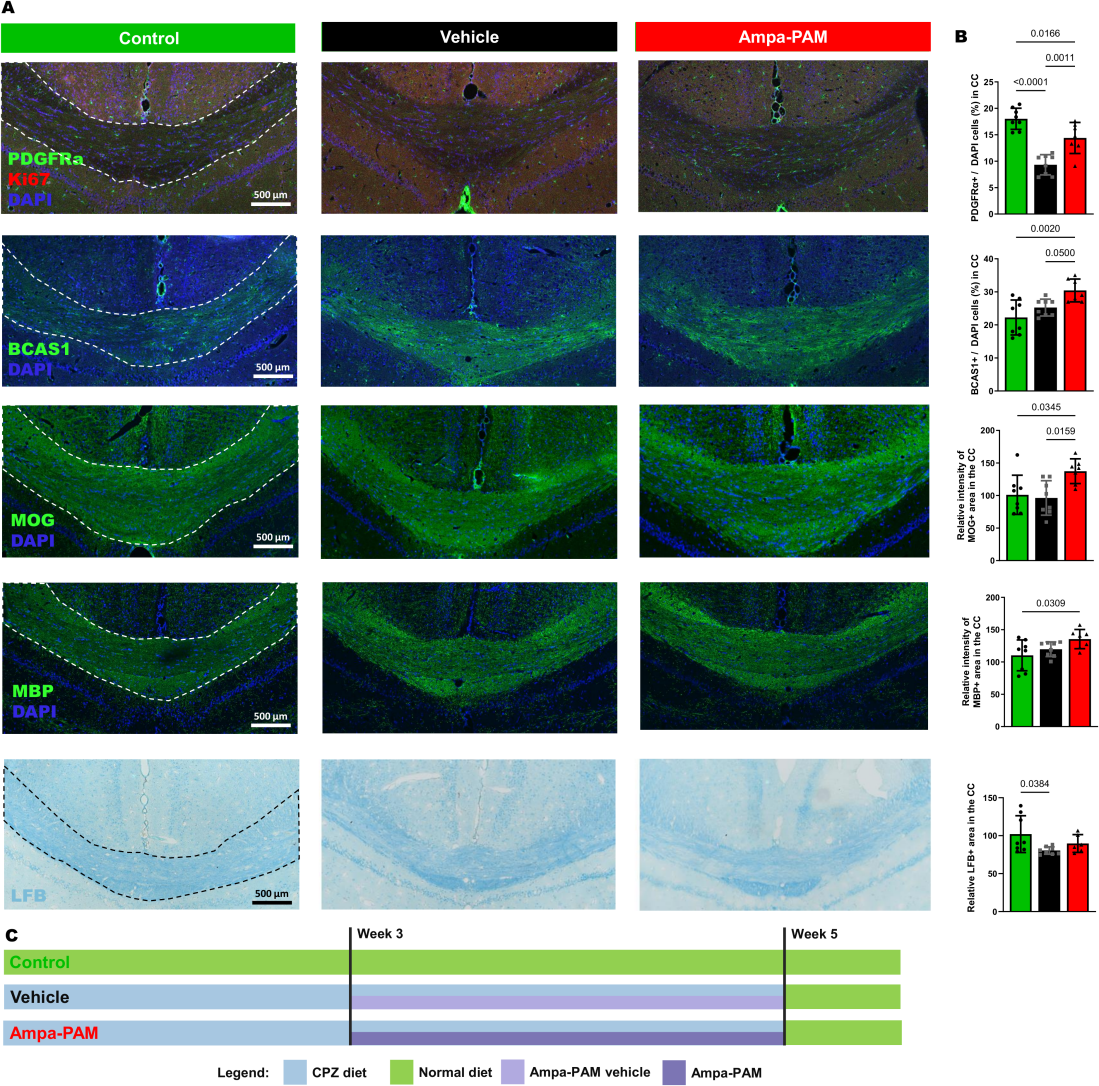
Remyelination marker in corpus callosum region post-Cuprizone treatment. Histological sections and quantitative analysis of remyelination in the corpus callosum are depicted, comparing vehicle and PF-4778574 treated animals. Expression levels of markers relevant for remyelination: PDGFRα/Ki-67, BCAS1, MOG, MBP and LFB was assessed in mice coronal brain sections. **(A)** provides representative images of these staining while the quantitative results are portrayed in **(B)**. **(C)** illustrates the demyelination and remyelination protocol using eight-week-old C57BL/6J mice divided into three groups: One fed control pellets (green, Control), and two fed 0.2% cuprizone pellets (black, Vehicle; red, AMPA-PAM). From week three, the AMPA-PAM group received daily PF-4778574 doses, while the Vehicle group received a vehicle. After six weeks of cuprizone, all mice switched to a control diet for one week to promote remyelination. Quantification was accomplished as described in section 2.2. All graphs represent the pooled mean ± SD (n=8 animals per group in total). The bar graph presents statistical significance, with p-values displayed over the bars, determined using ANOVA with Tukey`s *post hoc* test.

Cuprizone treatment protocol for remyelination study according to the protocol of 16, shortly: Eight-week-old C57BL/6J mice were used in the experiment and divided into three dietary groups. The first group was fed control pellets, while the second and third groups were fed 0.2% cuprizone-containing food pellets (Both SNIFF, Soest, Germany). Starting in the third week of treatment, mice in the AMPA-PAM group received daily doses of PF-4778574, while those in the vehicle group received an equivalent volume of vehicle. After six weeks of cuprizone treatment, all animals were switched to a control diet for one week to promote remyelination (**Figure 1C**).

We carried out a dose-finding study and a subsequent comparative/combination experiment to evaluate prophylactic and therapeutic administration of PF4778574 in both the EAE and cuprizone models. Based on a power calculation (G*Power version 3.1.9.7) aiming at a power (1-ß) of 0.8 and an alpha of 0.05 at an effect size of 1.5 and 1.4six animals per group were used in the EAE experiments and eight animals per group in the cuprizone model, respectively. The dose-finding study compared 0.1 mg/kg and 0.8 mg/kg PF4778574 (administered once daily) versus vehicle, incorporating regular OCT measurements on days 0 (baseline), 7, 14, 21, 35, 41, 56, 70, 84, and 112, with optomotor response (OMR) testing one day prior to each OCT session. In the subsequent comparative and combination study, OCT was performed on days 0, 42, and 85, while OMR assessments occurred on days 0, 7, 14, 21, 28, 42, 56, and 85. In both experiments, animals were assessed clinically on a daily basis. PF4778574 and fingolimod were administered orally each day at the doses described in **Table 1**.

**Table 1:** Treatment groups, therapy and treatment start.

Group name	Therapy and start of treatment (days post-immunization)
Sham+vehicle	DMSO: Cremophor:ddH2O (5:5:90), day 0
MOG+vehicle	DMSO: Cremophor:ddH2O (5:5:90), day 0
MOG+AMPA-PAM (d0)	0.1 mg/kg PF4778574, day 0
MOG+fingo (d0)	3 mg/kg fingolimod, day 0
MOG+AMPA-PAM (d14)	0.1 mg/kg PF4778574, day 14
MOG+AMPA-PAM(d0)+fingo (d0)	0.1 mg/kg PF4778574, day 0 + 3 mg/kg fingolimod, day 0

### 2.2 Euthanization and anesthesia protocol

For the OCT measurements, the animals were anesthetized using isoflurane, a widely used inhalational anesthetic known for its rapid induction and recovery properties. The anesthesia setup included a vaporizer provided by Harvard Apparatus Anesthetic Vaporizors, while the isoflurane itself was sourced from Piramal Critical Care. The induction phase was conducted at a concentration of 3.5% isoflurane to ensure a quick and effective loss of consciousness in the animals, followed by maintenance at a reduced concentration of 2% isoflurane to keep the animals in a stable anesthetized state throughout the procedure. A controlled oxygen flow rate of 0.6 liters per minute was employed to ensure adequate oxygenation during anesthesia. The anesthetic gas mixture was delivered directly to the mice through custom-designed nose cones, which allowed precise administration and minimized waste of the anesthetic agent.

Following the experimental procedures, the animals were humanely euthanized to ensure minimal suffering. This was achieved through intraperitoneal injection of a prepared solution containing 100 mg/kg of Ketamine and 20 mg/kg of Xylazine, dissolved in 250 µl of 0.9% NaCl saline solution. This combination is widely recognized for its effectiveness in humane animal euthanasia. Cardiac perfusion was subsequently performed. This involved flushing the circulatory system with phosphate-buffered saline (PBS) obtained from Gibco (Carlsbad, USA) to remove blood and prepare tissues for downstream analyses. These steps were carried out in strict adherence to ethical guidelines and protocols for animal experimentation to ensure the welfare of the animals and the reliability of the experimental results as mentioned in point 11.

### 2.3 OCT, OMR and other outcome measures

OCT assessments were performed as previously described and are reported in line with the APOSTEL reporting guidelines (17, 18). The OCT-based primary outcome parameters included the thickness of the inner retinal layers, total retinal thickness, and outer retinal layers as evaluated in volume scans around the optic disc. During OCT measurements, the animals were anesthetized as described in point 2.2.

We carried out regular OCT measurements using the Spectralis^®^ HRA + OCT instrument (Heidelberg Engineering, Germany), which was modified for rodent use as previously explained (19). The Heidelberg Eye Explorer software was utilized to segment retinal volume scans, with manual correction for any segmentation errors. The volume of the area around the optic disc was evaluated using the ETDRS grid, avoiding the center with the disc, as previously documented (20). Periodic examinations were made of the inner retinal layer (IRL), outer retinal layer (ORL), and total retinal thickness (TRT), and were compared with baseline measurements. The IRL layer includes: NFL, GCL and IPL whereas the ORL layer contains: INL, OPL, ONL, ELM, IS/OS, RPE and Choroid. The TRT consists of both: IRL + ORL.

The spatial frequency measurement via OMR was taken as a primary functional output (21). We carried out OMR measurements at periodic intervals using the OptoMotry^®^ system from Cerebral Mechanics, simultaneously with OCT and cSLO measurements. The parameter for visual function was the spatial frequency, which was identified by varying the spatial frequency randomly to determine the threshold where the mouse could track, as reported earlier (20, 21).

Other histology-based parameters were assessed as secondary outcome criteria. A standardized scoring system was employed to evaluate the severity of EAE symptoms. In the EAE model, disease severity is monitored using a clinical scoring system from 0.5 to 5, where 0.5 indicates general weakness and fatigue, 1 indicates complete tail paralysis without other neurological deficits, 1.5 adds a restricted righting reflex, 2 (weak) and 2.5 (severe) further introduces partial hind limb paralysis, 3 indicates reversible complete hind limb paralysis, 3.5 includes a ≥20% reduction in body weight, 4 adds partial forelimb paralysis, and 5 denotes a moribund animal or death due to EAE.

### 2.4 Histological analysis

After euthanization of the mice as described in point 2.2, the optic nerves were then isolated, fixed overnight in 4% paraformaldehyde (Carl Roth, Karlsruhe, Germany), and later treated with a sucrose gradient for dehydration and embedded in O.C.T. compound (Sakura™ Finetek, Alphen aan den Rijn, Netherlands).

Optic nerve: Longitudinal optic nerve slices of five micrometers were obtained for fluorescence staining. Cross-sectional slices of the optic nerves were utilized to quantify and evaluate the condition of myelination using antibodies against myelin basic protein (MBP) (Millipore, #MAB386, 1:500). Microglial activation was assessed using antibodies against Iba1 (Wako, #019-19741, 1:500) under a Leica DMi8 confocal microscope equipped with a Leica HyD detector (63x objective lens magnification). The secondary antibodies used were goat anti-mouse Cy 2, goat anti-rabbit Cy 3, goat ant-rat Cy3 (Millipore, 1:500) and Alexa Fluor™ 488 rabbit anti-mouse (Invitrogen, 1:500). ImageJ software was utilized to analyze the numbers of cells stained with Iba1, expressed as a ratio to DAPI staining, and the overall signal for MBP (positive total area in the red channel).

Retinal whole mount: To further examine the retina, a semi-automated count of Brn3a+ cells on retinal flatmounts was performed. The retinae were stained with Brn3a (Santa Cruz Biotechnology, #sc-8429, 1:200) antibody and subsequently flat-mounted on glass slides. The secondary antibodies used was Alexa Fluor™ 488 rabbit anti-mouse (Invitrogen, 1:500). Each retina was partitioned into four quadrants, each with three areas: central, mid-periphery, and far-periphery. For each eye, the total count of Brn3a+ cells were calculated by aggregating the counts from all 12 imaged areas.

Brain: Brain cross sections were obtained for fluorescence staining to quantify myelination status and OPC´s activation. OPC´s were detected using antibodies against PDGFRα (ThermoFisher Scientific, #A21207, 1:250). Cell proliferation was detected using antibodies against Ki-67 (AbCam, #16667, 1:250). MOG was detected using antibodies at 1:500 dilution (Millipore, #8-81805). MBP was detected using antibodies at 1:250 dilution (Bio-Rad, #MCA409S). BCAS1 was detected using antibodies at 1:250 dilution (Santa Cruz, #10839529). The secondary antibodies used were goat anti-rat Cy3 (Millipore, 1:500) and Alexa Fluor™ 488 goat anti-mouse (Invitrogen, 1:250). Further, slides containing 12-μm coronal section were incubated overnight in LFB (Sigma-Aldrich) solution (0.1% LFB, 4% glacial acetic acid in 96% ethanol) at 56°C. Afterward, redundant LFB staining was washed out using 0.05% lithium carbonate solution (in ddH2O), tissue was dehydrated and embedded in ROTI-Histokit II (Carl Roth, Karlsruhe, Germany). Quantification was performed as follows: For PDGFRα and BCAS1, the number of positive cells was counted and expressed as a ratio relative to DAPI-positive cells within the marked corpus callosum area. For MOG and MBP, the relative intensity of the MOG and/or MBP-positive regions was measured within the marked corpus callosum area after normalization. Similarly, for LFB, the relative area of LFB positivity within the marked corpus callosum region was determined following normalization.

### 2.5 Data analysis

Prism (version 9, Graphpad Software, Inc.) and IBM SPSS Statistics (version 20, IBM Corporation, USA) were used for statistical analysis. The data of retinal parameters were analyzed using generalized estimation equation (GEE) models. The Dunnett’s *post hoc* test, in conjunction with a one-way ANOVA, was used for group means analysis, except for the corpus callosum analyses, which were evaluated using Tukey’s test.

For OCT data analysis, we followed our previously published approaches for evaluating EAE outcomes, which provide detailed guidance on handling measurements from both eyes in a statistically appropriate manner (17, 22). Briefly, we first applied the OCT device software’s automated segmentation to derive thickness values for relevant retinal layers; afterward, we performed manual correction of any segmentation errors while ensuring the investigator was blinded to experimental groups. We then used the 1, 2, and 3 mm ETDRS grid to calculate mean thickness values, excluding the inner 1 mm region containing the optic disc, which typically covered an angle of approximately 25°. All scans were evaluated based on predefined quality cutoffs (e.g., >20 decibels) to maintain consistency across samples. For statistical analysis, we employed generalized estimating equation models using an exchangeable working correlation matrix accounting for within-subject inter-eye correlations (), to ensure valid inferences from animals in which both eyes were assessed (17, 18, 22).

For OMR data analysis, we also incorporated methodologies outlined in our earlier work (22), with attention to the potential dependence of measurements from both eyes. In this setup, the software randomly changed the direction of the moving grid, and the tracking response (clockwise vs. counterclockwise) was used to determine whether the animal followed the left or right eye stimulus. Step sizes of the stimulus were either adjusted manually or automatically through the software’s adaptive feature. To maintain robust engagement throughout the testing session, we stimulated the animals with whistling sounds and sporadic screen blanking. At the end of each trial, the summary data were exported for further processing, and statistical analyses again utilized the GEE models outlined above.

## 3 Results

### 3.1 Dose finding study

In our study aimed at evaluating the efficacy of PF4778574 in preventing acute inflammatory exacerbations and chronic degeneration, we conducted a 110-day longitudinal analysis of retinal neurodegeneration using the MOG33-55 peptide-induced EAE model in C57BL/6J mice. We examined the effects of two different concentrations of PF4778574 (0.1 and 0.8 mg/kg bodyweight, administered subcutaneously), starting the treatment at the same time as the immunization (prophylactic treatment), comparing treated animals with untreated EAE mice (MOG+vehicle) and sham non-EAE mice (sham+vehicle).

**Figure 2:**
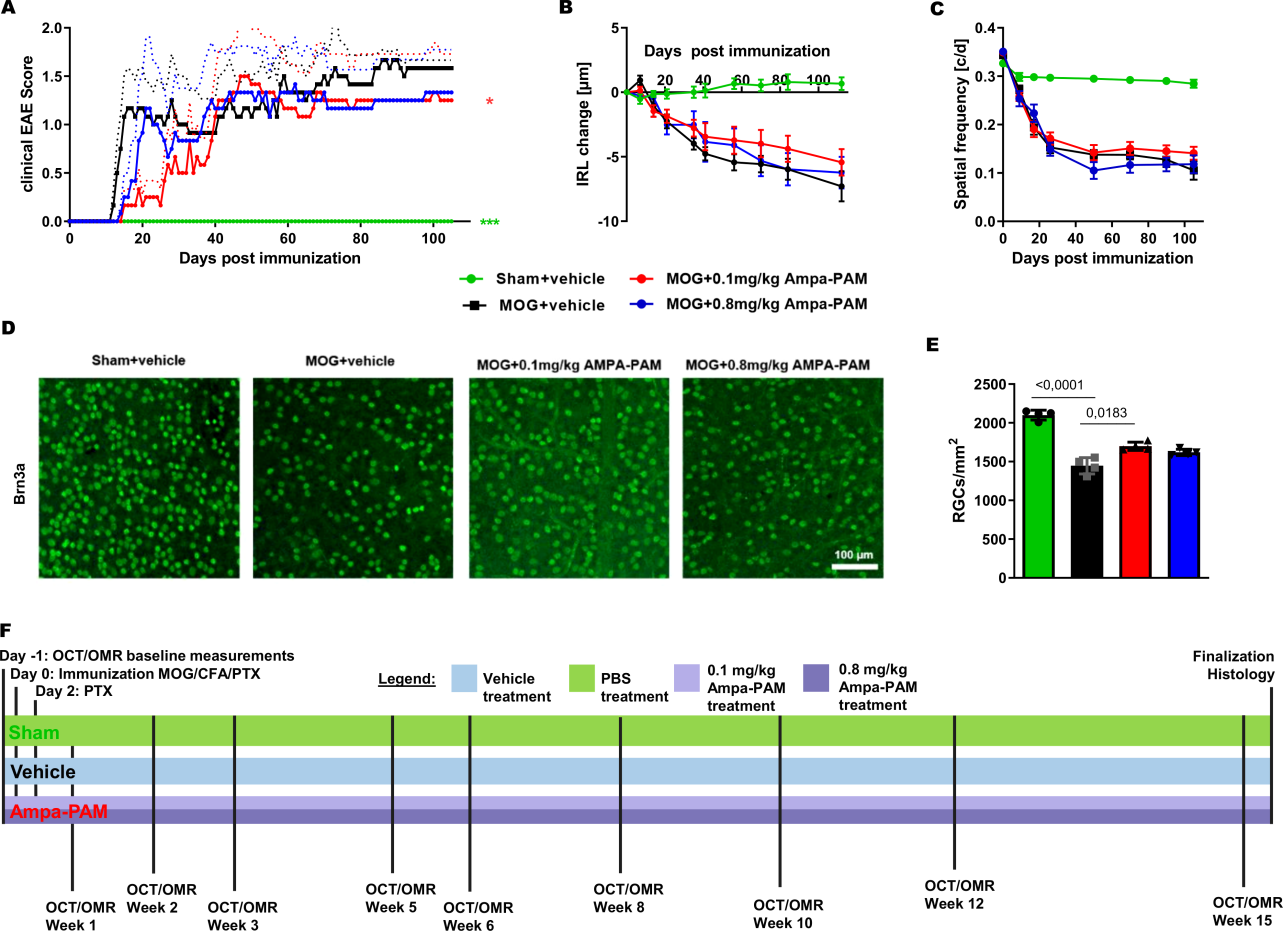
AMPA-PAM (PF4778574) attenuates MOG35-55-induced EAE in C57BL/6J mice in a dose dependent manner. **(A)** Clinical EAE score, **(B)** degeneration of the inner retinal layers and **(C)** visual function by spatial frequency in cycles per degree (c/d) of female C57BL/6J EAE mice over 110 days of EAE. **(D)** Brn3a stained RGCs after 110 days of EAE of Sham, MOG EAE and AMPA-PAM treated mice. **(E)** The bar graph shows the RGC density 110 days after immunization. AMPA-PAM was administered on the day of immunization. **(F)** Illustrates the treatment and evaluation regimen of the EAE experiment. Graphs A-C represent the pooled mean ± SEM (out of two independent experiments each with n = 6 animals per group), with *p<0.05, AUC compared by ANOVA with LSD *post hoc* test for time course compared to untreated MOG EAE. The bar graph E presents statistical significance, with p-values displayed over the bars, determined using ANOVA with Dunnett’s *post hoc* test, compared to MOG EAE untreated mice.

The prophylactic administration of 0.1 mg/kg PF4778574 had a significant positive impact on the clinical EAE scores (Mean Difference [MD]: 0.5487, p<0.05), whereas the higher dose of 0.8 mg/kg did not result in a statistically significant effect (MD: 0.3303, not significant [ns]), as shown in **Figure 2A**.

When examining structural changes in the retina via OCT measurements, untreated sham control mice maintained a stable inner retinal layer (IRL) thickness throughout the study period. In contrast, mice immunized with the MOG peptide exhibited a significant reduction in IRL thickness (MD: 3.740, p<0.001), which persisted up to day 110. The 0.1 mg/kg PF4778574 treatment significantly slowed the degeneration of the IRL (MD: 0.8487, p<0.05) compared to vehicle-treated MOG EAE mice (**Figure 2B**). However, the 0.8 mg/kg dose did not significantly affect IRL degeneration (MD: 0.4244, ns).

Furthermore, PF4778574 did not show a significant benefit in preserving visual acuity, as both the 0.1 mg/kg (MD: 0.009406) and 0.8 mg/kg (MD: 0.004781) doses failed to produce statistically significant improvements compared to vehicle-treated MOG EAE mice (**Figure 2C**).

Immunohistological staining of retinal wholemounts revealed that untreated EAE mice experienced substantial retinal ganglion cell (RGC) loss, as shown in **Figures 2D, E**. The 0.1 mg/kg dose of PF4778574 modestly reduced this loss (MD: 252.8, p<0.05), whereas the 0.8 mg/kg dose did not yield a significant effect (MD: 173.0, ns). These findings align with the EAE clinical scores. **Figure 6F** shows the treatment and readout intervals.

**Figure 3:**
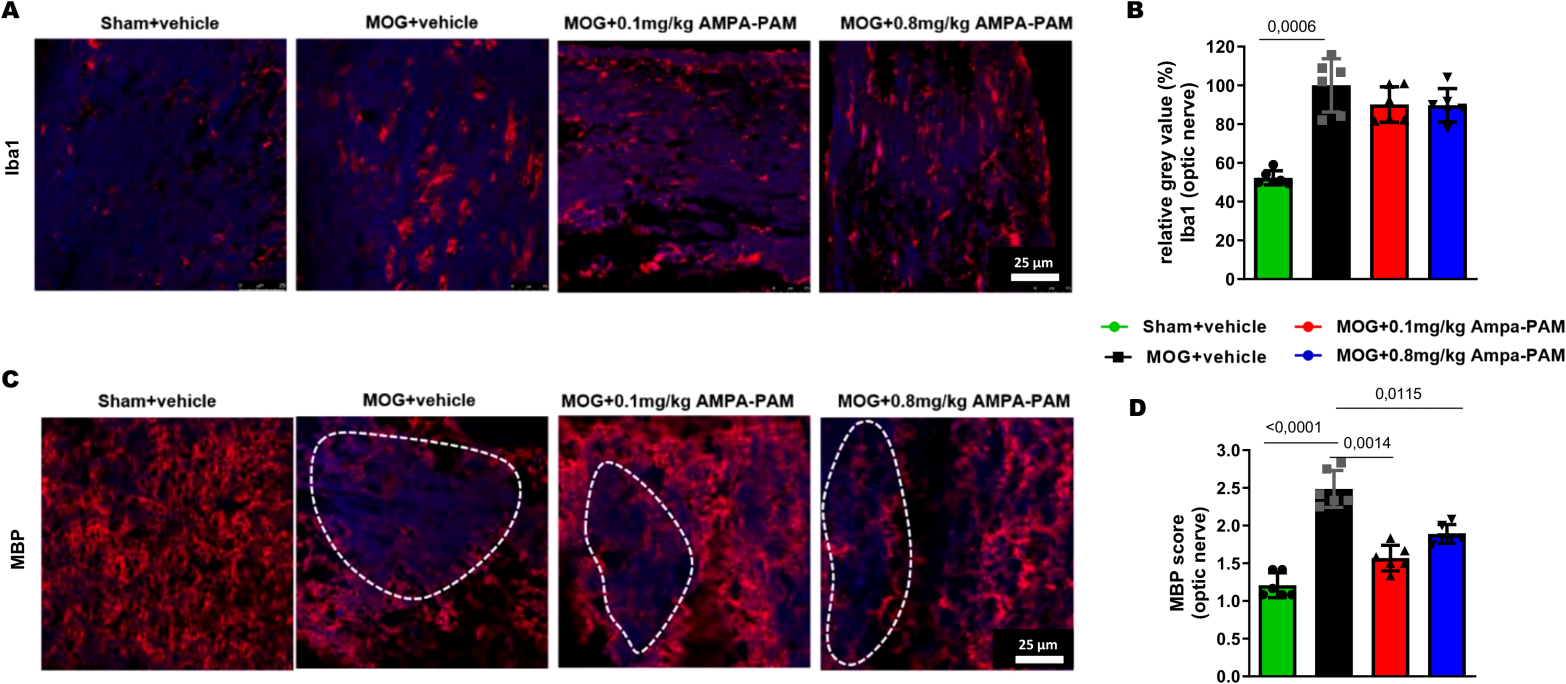
Prophylactic AMPA-PAM (PF4778574) therapy attenuates optic nerve demyelination. Longitudinal sections of optic nerves of C57Bl/6J mice were stained for **(A)** Iba1 and **(C)** MBP 110 days after MOG35-55 immunization; dotted lines indicate areas of demyelination. Quantitative analyses of microglial activation (Iba1+ cells) **(B)** by fluorescence intensity measurement and myelin status (MBP intensity score) **(D)**. One optic nerve per mouse was included. All graphs represent the pooled mean ± SEM (n = 6 animals per group out of two independent experiments). The bar graph presents statistical significance, with p-values displayed over the bars, determined using ANOVA with Dunnett’s *post hoc* test, compared to MOG EAE untreated mice.

To assess the impact of PF4778574 on immune cell infiltration into the CNS during EAE, we performed histological analysis of Iba1+ microglia in longitudinal sections of the optic nerve (**Figure 3A**). Neither the 0.1 mg/kg (MD: 9.894, ns) nor the 0.8 mg/kg (MD: 10.27, ns) prophylactic treatments resulted in a significant reduction in microglial activity in the optic nerves compared to untreated EAE mice (**Figure 3B**).

We also evaluated the myelin integrity of the optic nerve by performing immunohistological staining for Myelin Basic Protein (MBP). MOG-immunized mice showed extensive demyelination in the optic nerve, whereas untreated sham mice exhibited uniform myelin structure (MD: 1.278, p<0.001). Treatment with PF4778574 led to a significant reduction in the extent of lesions in the MBP-stained optic nerves at both the 0.1 mg/kg (MD: 0.8333, p<0.01) and 0.8 mg/kg (MD: 0.5833, p<0.05) doses (**Figures 3C, D**).

### 3.2 Comparative and combinatory study

In the subsequent phase of our study, we combined the dose of PF4778574 that demonstrated the highest efficacy in the earlier dose-response experiment (0.1 mg/kg) with the well-established immunomodulatory agent, fingolimod, at 3 mg/kg (administered orally) in the EAE model. The treatment protocols used are outlined in **Table 1**.

**Figure 4:**
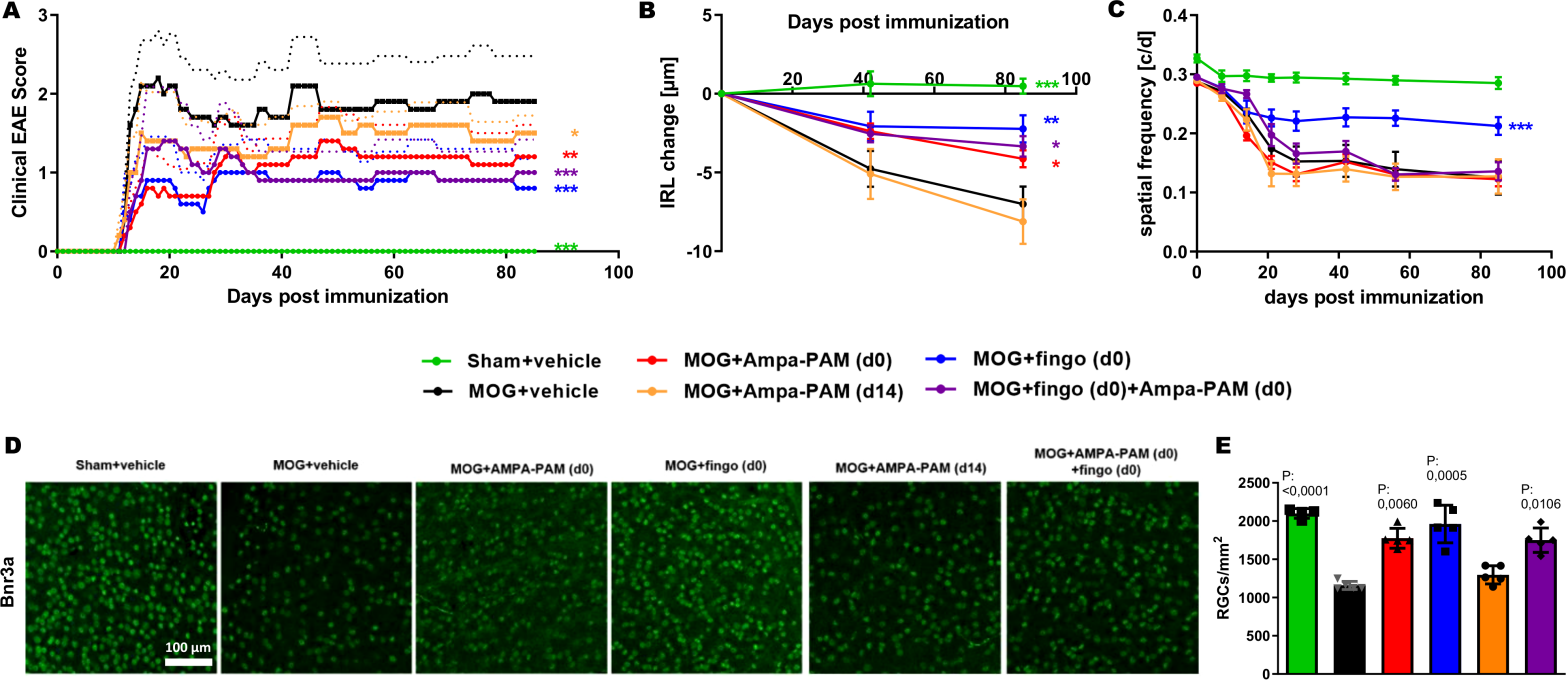
PF4778574 and fingolimod therapy attenuate MOG35-55-induced EAE in C57BL/6J mice. **(A)** Clinical EAE score, **(B)** degeneration of the inner retinal layers and **(C)** visual function by spatial frequency in cycles per degree (c/d) of female C57BL/6J EAE mice over 90 days of EAE. **(D)** Brn3a stained RGCs after 90 days of EAE of Sham, MOG EAE and AMPA-PAM treated mice. **(E)** The bar graph shows the RGC density 90 days after immunization. AMPA-PAM was administered on the day of immunization. All graphs represent the pooled mean ± SEM (n = 6 animals per group), with *p<0.05, **p<0.01, ***p<0.001, area under the curve compared by ANOVA with Dunnett’s *post hoc* test for time course compared to untreated MOG EAE. The graphs present statistical significance, with p-values displayed over the bars, determined using ANOVA with Dunnett’s *post hoc* test, compared to MOG EAE untreated mice.

Both prophylactic treatment with fingolimod (MD: 0.8279, p<0.001) and PF4778574 (MD: 0.6605, p<0.01) significantly reduced the clinical EAE scores. Even when PF4778574 treatment was delayed and initiated therapeutically 14 days post-immunization, we observed a notable improvement in clinical disability in EAE mice (MD: 0.3488, p<0.05) compared to the untreated MOG group. However, combining PF4778574 with fingolimod did not result in any synergistic effects beyond what was achieved with fingolimod (fingo) alone (MD: 0.7337, p<0.001). These findings from the EAE clinical scores were supported by OCT evaluations of the inner retinal layer (IRL) thickness (MD: Fingo D0 = 2.493, p<0.01; AMPA-PAM D0 = 1.750, p<0.05; Fingo D0 & AMPA-PAM D0 = 1.963, p<0.05), except in the case of delayed PF4778574 monotherapy, which did not show a significant difference compared to the vehicle treated MOG control (**Figures 4A, B**).

Functionally, only fingolimod monotherapy significantly improved visual acuity (MD: 0.04798, p<0.001), while PF4778574 (MD: 0.01261, ns) and all combination therapies did not significantly alter visual function in EAE mice (**Figure 4C**). Histologically, retinal ganglion cell (RGC) loss after 90 days of EAE was reduced by both prophylactic PF4778574 (MD: 609.5, p<0.01) and prophylactic fingolimod (MD: 813.0, p<0.001), as well as by the combination therapy (MD: 565.1, p<0.05) (**Figures 4D, E**).

**Figure 5:**
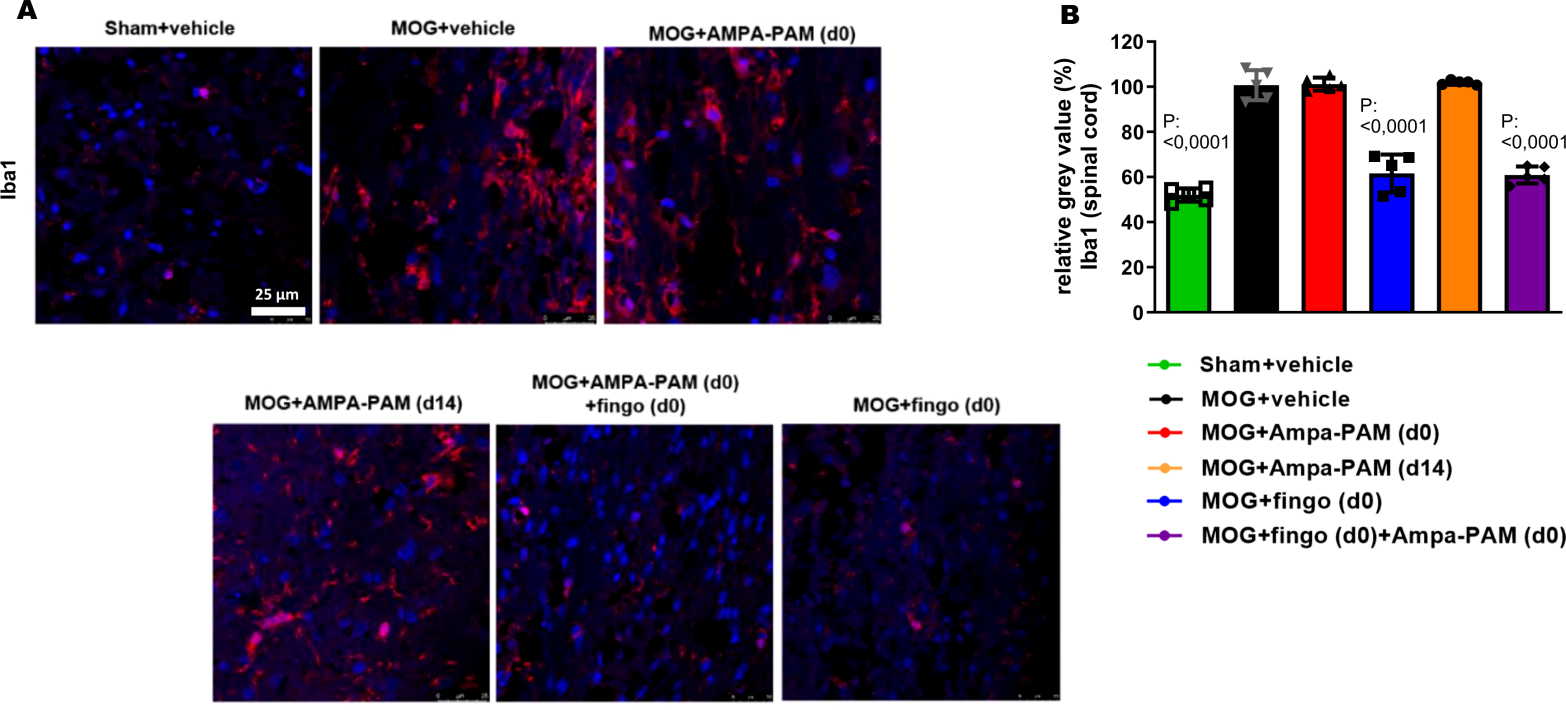
PF4778574 does not reduce the number of Iba1+ infiltrates into the spinal cord of EAE mice. **(A)** Longitudinal sections of spinal cord of C57Bl/6J mice were stained for Iba1 90 days after MOG35-55 immunization; dotted lines indicate areas of demyelination. **(B)** Quantitative analyses of microglial activation (Iba1) by fluorescence intensity measurement. All graphs represent the pooled mean ± SEM (n = 6 animals per group). The bar graph presents statistical significance, with p-values displayed over the bars, determined using ANOVA with Dunnett’s *post hoc* test, compared to MOG EAE untreated mice.

Further histological analysis of the spinal cord 90 days post-MOG35-55 immunization revealed that neither prophylactic nor therapeutic PF4778574 monotherapy reduced the number of Iba1+ microglia. However, both fingolimod monotherapy (MD: 37.60, p<0.001) and the combination therapy (MD: 38.67, p<0.001) significantly decreased the number of these microglial infiltrates compared to untreated EAE mice (**Figures 5A, B**).

**Figure 6:**
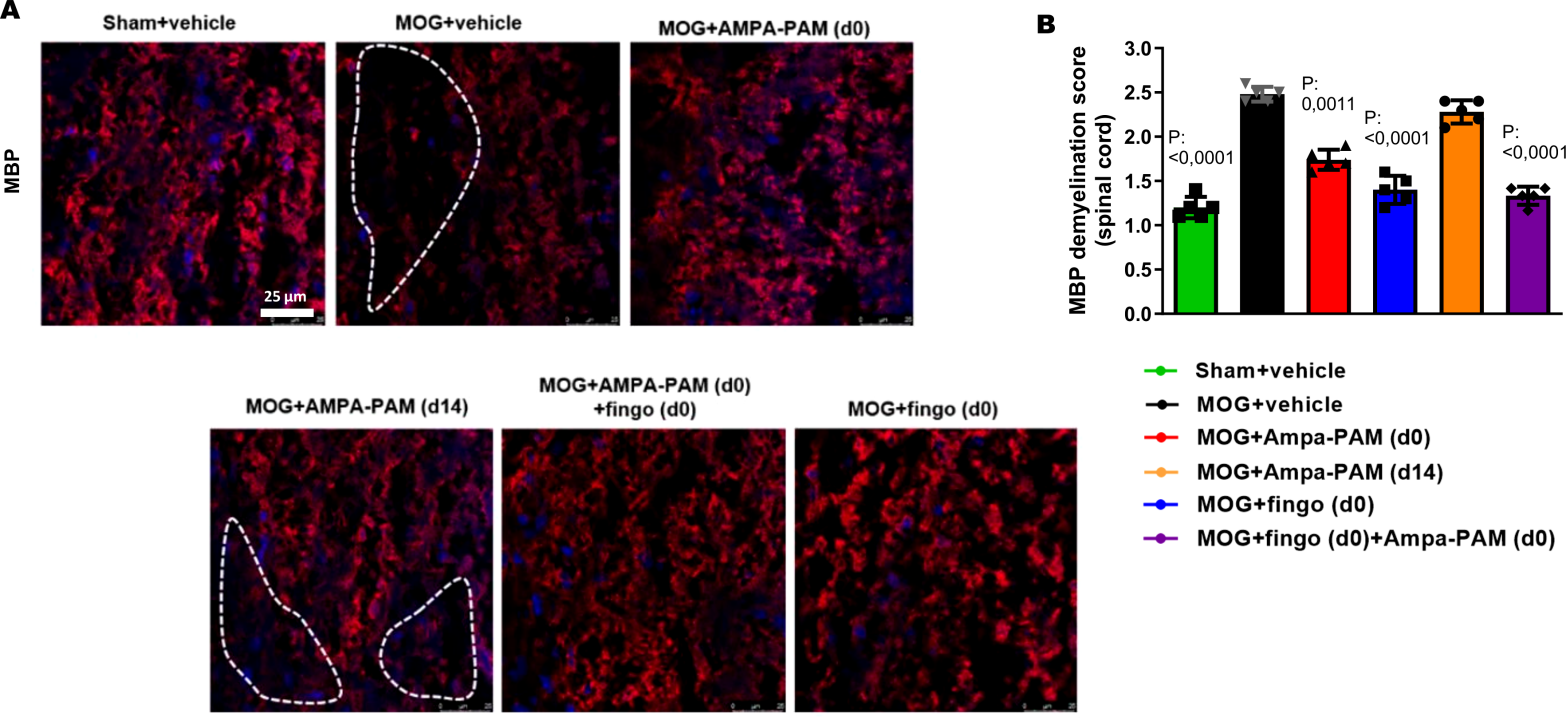
Prophylactic PF4778574 and fingolimod treatment reduce demyelination of the spinal cord in EAE mice. **(A)** Longitudinal sections of spinal cord of C57Bl/6J mice were stained for MBP 90 days after MOG35-55 immunization; dotted lines indicate areas of demyelination. **(B)** Quantitative analyses of the myelin status (MBP demyelination score). All graphs represent the pooled mean ± SEM (n = 6 animals per group). The bar graph presents statistical significance, with p-values displayed over the bars, determined using ANOVA with Dunnett’s *post hoc* test, compared to MOG EAE untreated mice.

To assess the protective effects against demyelination in the spinal cord, MBP staining was performed. It showed that both prophylactic PF4778574 (MD: 0.7333, p<0.01) and fingolimod (MD: 1.067, p<0.001) monotherapy, as well as the combination therapy (MD: 1.100, p<0.01), significantly reduced the extent of demyelination in the EAE model compared to the untreated MOG cohort 90 days post-immunization. However, therapeutic PF4778574 intervention did not significantly reduce lesion areas in the spinal cord (MD: 0.6333, ns) (**Figures 6A, B**).

To further assess the remyelinating potential of PF4778574, we used the Cuprizone model as described in point 2.1., focusing on histological sections of the corpus callosum (**Figure 1A**). PF4778574 treatment led to a significant increase in PDGFRα-positive cells (MD: 5.064, p<0.01) compared to vehicle-treated animals, indicating an enhanced presence of oligodendrocyte precursor cells (OPCs) crucial for myelin development and regeneration. BCAS1 staining revealed a significant rise in late-stage oligodendrocyte lineage cells involved in active myelination processes in the PF4778574-treated group (MD: 5.179, p<0.05). Additionally, PF4778574 treatment resulted in a higher relative intensity of the MOG-positive area (MD: 41.14, p<0.05), suggesting improved myelin integrity. While MBP staining (MD: 15.73, ns) and LFB staining (MD: 9.229, ns) did not show statistically significant differences, both exhibited trends toward higher expression in the treated group (**Figure 1B**). **Figure 1C** illustrates the diet and treatment regimen. MBP is an essential protein reflecting the extent of myelination, and LFB is a staining technique that highlights overall myelin content. These observations suggest that PF4778574 may enhance remyelination, even if some marker-quantification did not reach statistical significance.

## 4 Discussion

In the present study, we aimed to systematically investigate the therapeutic and preventive effects of the type-II AMPA-PAM PF4778574 as well as a combination therapy of PF4778574 and fingolimod, - as an example for an approved immunomodulatory treatment for MS - on EAEON, an established experimental rodent model of MS optic neuritis. To conduct a comprehensive assessment, we utilized a multifaceted approach, incorporating both clinical and experimental measures. Our evaluation included clinical EAE scoring, which served to assess the severity of clinical manifestations in the disease model. Further, we incorporated OCT and OMR evaluations, established *in-vivo* readout methodologies in the examination of visual function and structural integrity in the context of optic neuritis. Finally, we performed histological evaluations, which provided insights into the cellular and structural changes within the CNS, thereby complementing our clinical observations with a deeper understanding of the underlying pathological alterations.

The role of excitotoxicity, where overactive glutamate receptors lead to neuronal damage, is documented in MS (12). This study’s focus is on AMPA-PAMs, which modulate AMPA receptors to prevent over-activation and excitotoxicity, therefore providing a promising aspect of investigation in the context of MS pathology. Additionally, the mode of action of AMPA-PAMs could also involve enhancing synaptic transmission in cases of neuronal dysfunction, which results from demyelination and degeneration in the CNS. This dual potential—preventing excitotoxic damage while supporting synaptic function—underscores the therapeutic promise of AMPA-PAMs in managing MS.

A central observation from this study is the successful mitigation of clinical disability in the EAE model following both prophylactic and therapeutic administration of the AMPA-PAM PF4778574. However, the lack of impact on Iba1+ microglia contrasts with prior findings that AMPA receptor modulation might also have potential anti-inflammatory effects (13–15). Of note, these previous studies investigated the effect of AMPA-antagonists, rather than AMPA-PAMs, which act in a different pattern on the AMPA receptors than PAMs. This disparity suggests that while AMPA-PAMs like PF4778574 may influence synaptic and neuronal function and/or excitotoxicity, they may not significantly impact neuroinflammation, the essential primary process in MS which can very effectively be targeted by approved therapeutics like the fingolimod used in our study.

We observed an enhanced myelin integrity in the optic nerve, corpus callosum and spinal cord following prophylactic AMPA-PAM treatment. Furthermore, AMPA-PAM treatment significantly reduced the loss of retinal ganglion during optic neuritis. This finding aligns with recent studies which highlight the potential neuroprotective roles of AMPA-PAMs beyond merely neurotransmission (23). Such effects could have significant therapeutic implications in neuroinflammatory or degenerative conditions where myelin loss is prominent.

The lack of additive benefits and diminished efficacy in combined treatment regimens involving PF4778574 and fingolimod was disappointing. Potential reason may have been drug interactions as both substances undergo metabolism by cytochrome P450 (CYP) (24). Further investigations to understand this interaction could offer insights into optimizing combination treatments involving AMPA-PAMs and existing MS therapies.

Interestingly, the lower dose of PF4778574 in our study contributed to a better outcome compared to the higher dose. Such a bell shaped dose response curve is characteristic for hormesis, which is classically observed when doses above a certain threshold lead to harmful or toxic effects (25) and has been observed in the context of other remyelinating therapeutics (20). The typical bell-shaped dose-response curve is thought to arise from the balance between beneficial adaptive responses and potential toxicity at higher doses (26). However, regarding PF4778574 specifically, the typical adverse effects are seizures. We carefully adjusted the treatment dose below the seizure threshold and observed no seizures during our experiments. Thus, harmful effects resulting from the high PF4778574 dose in our experiments might result from a sub-seizure threshold overstimulation of AMPA receptors, potentially leading to a mild form of excitotoxicity (27). This might explain why the lower dose was more beneficial in the EAE model.

While we did not directly measure the electrical activity of retinal ganglion cells or optic nerve conduction speed (e.g., via pattern electroretinography [pERG] or visual evoked potentials [VEP]), these techniques could provide valuable insights into whether PF4778574 influences neuronal excitability *in vivo*. Future studies incorporating pERG or VEP could help clarify any dose-dependent effects on RGC function and optic nerve signal transmission, thereby offering a more complete understanding of how AMPA-PAM treatment modulates neuronal activity in the context of MS.

PF4778574 did not impact microglial activity. Thus, it is plausible to assume that the treatments benefits mainly result from functional and structural protection of the neuronal structures rather than inhibiting neuroinflammation. This is consistent with the role of AMPA receptors in neurotransmission and synaptic plasticity (28).

Reduced myelin damage observed with both doses of PF4778574 at the endpoint suggests possible myelin-protective and/or remyelinating effects. These effects might indirectly result from modified neuronal activity, which could affect the local environment and support myelin maintenance and/or remyelination (29, 30). Our results in the cuprizone-induced demyelination model suggest that PF4778574 may indeed enhance remyelination after toxic demyelination, as indicated by increased numbers of oligodendrocyte progenitor cells (OPCs) and increased myelin content within the corpus callosum, as shown by PDGFRα, MOG, and BCAS1 staining, respectively. However, other myelin markers, MBP and LFB, showed only positive trends for remyelinating effects of PF4778574 but did not reach statistical significance. In these short-term cuprizone demyelination experiments, the timing of de- and remyelination is crucial. Further studies with larger sample sizes are needed to evaluate the remyelinating capacity of PF4778574 and its mode of action.

**Figure 7:**
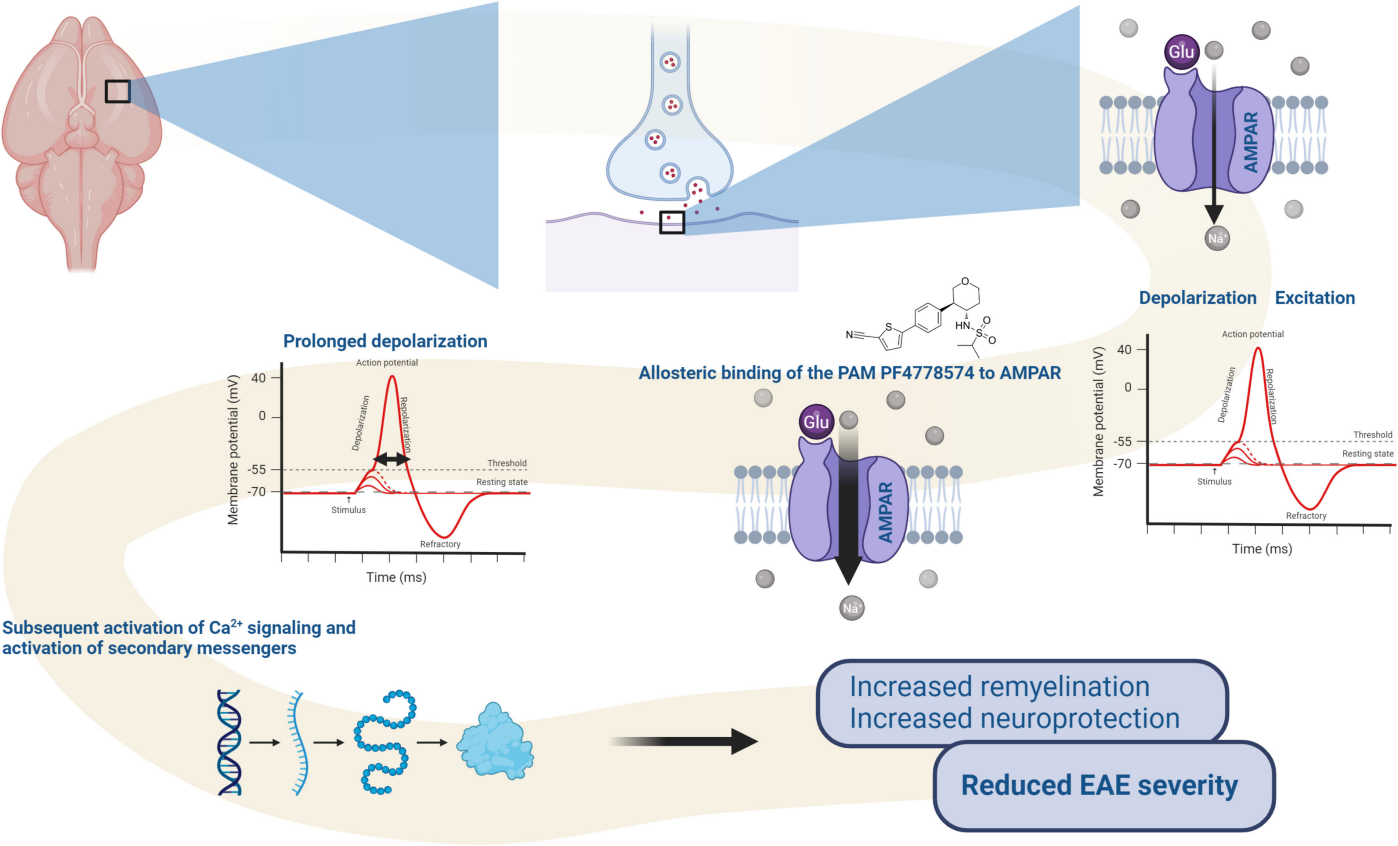
Proposed mode of action of PF-4778574: Upon allosteric binding to AMPA receptors, the PAM enhances glutamate-induced sodium (Na^+^) influx, leading to prolonged depolarization. This sustained depolarization increases the likelihood of activating voltage-gated calcium channels, which in turn triggers calcium (Ca^2+^) signaling and the activation of secondary messenger pathways. These processes promote the activation of oligodendrocyte precursor cells (OPCs), facilitating remyelination and contributing to the reduced severity of EAE symptoms. The diagram highlights the impact of AMPA-PAM on synaptic activity, neural signaling, and therapeutic outcomes in demyelinating conditions.

In conclusion, PF4778574 reduces clinical disability in the context of EAE following both prophylactic and therapeutic administration. Improvements in EAE scores were evaluated by structural measurements of the inner retinal layer on OCT and assessments of retinal ganglion cell counts. However, PF4778574 did not affect the number of Iba1+ infiltrates in the optic nerve and spinal cord. Nevertheless, prophylactic AMPA-PAM treatment enhanced myelin integrity. Interestingly, combination treatment with fingolimod did not produce additive benefits and showed diminished efficacy compared to monotherapies. This could potentially be attributed to drug interactions, given that both substances are metabolized by cytochrome P450 enzymes (CYP), and induction of specific CYPs may influence the efficacy of fingolimod. This study adds to the literature exploring the potential of AMPA-PAMs in degenerative and neuroinflammatory diseases such as MS. These observations, as illustrated in the schematic representation in **Figure 7**, underscore the potential therapeutic benefits of AMPA-PAM. And demonstrates the capacity to regulate neural activity and support the maintenance of myelin integrity. As PF4778574 is well-tolerated and safe, it may be a suitable candidate for further investigation in clinical trials.

## Data Availability

The raw data supporting the conclusions of this article will be made available by the authors, without undue reservation.

## Glossary

4AP: 4-Aminopyridine
AMPA-PAM: AMPA-type glutamate receptor Positive Allosteric Modulator
AMPAR: AMPA Receptor
ANOVA: Analysis of Variance
BDNF: Brain-Derived Neurotrophic Factor
CFA: Complete Freund’s Adjuvant
CNS: Central Nervous System
Cuprizone (CPZ): Copper Chelating Agent used to induce demyelination
CYP: Cytochrome P450
DAPI: 4′,6-Diamidin-2-phenylindol
EAE: Experimental Autoimmune Encephalomyelitis
ELM: External Limiting Membrane
fEPSP: Field Excitatory Postsynaptic Potential
GCL: Ganglion Cell Layer
GEE: Generalized Estimation Equation
Iba1: Ionized Calcium Binding Adapter Molecule 1
INL: Inner Nuclear Layer
IPL: Inner Plexiform Layer
IRL: Inner Retinal Layer
IS/OS: Inner and Outer Segments of Photoreceptors
Ki-67: Proliferation Marker Protein
LFB: Luxol Fast Blue
MBP: Myelin Basic Protein
MOG35-55: Myelin Oligodendrocyte Glycoprotein fragment 35-55
MS: Multiple Sclerosis
NFL: Nerve Fiber Layer
OCT: Optical Coherence Tomography
OMR: Optomotor Response
ONL: Outer Nuclear Layer
OPC: Oligodendrocyte Progenitor Cell
OPL: Outer Plexiform Layer
ORL: Outer Retinal Layer
PDGFRα: Platelet-Derived Growth Factor Receptor Alpha
PTX: Pertussis Toxin
RGC: Retinal Ganglion Cell
RPE: Retinal Pigment Epithelium
TRT: Total Retinal Thickness
